# Vacuolar proteomic analysis reveals tonoplast transporters for accumulation of citric acid and sugar in citrus fruit

**DOI:** 10.1093/hr/uhad249

**Published:** 2023-11-28

**Authors:** Zuolin Mao, Yue Wang, Mengdi Li, Shuhang Zhang, Zeqi Zhao, Qiang Xu, Ji-Hong Liu, Chunlong Li

**Affiliations:** National Key Laboratory for Germplasm Innovation & Utilization of Horticultural Crops, College of Horticulture and Forestry Sciences, Huazhong Agricultural University, Wuhan 430070, China; National Key Laboratory for Germplasm Innovation & Utilization of Horticultural Crops, College of Horticulture and Forestry Sciences, Huazhong Agricultural University, Wuhan 430070, China; National Key Laboratory for Germplasm Innovation & Utilization of Horticultural Crops, College of Horticulture and Forestry Sciences, Huazhong Agricultural University, Wuhan 430070, China; National Key Laboratory for Germplasm Innovation & Utilization of Horticultural Crops, College of Horticulture and Forestry Sciences, Huazhong Agricultural University, Wuhan 430070, China; National Key Laboratory for Germplasm Innovation & Utilization of Horticultural Crops, College of Horticulture and Forestry Sciences, Huazhong Agricultural University, Wuhan 430070, China; National Key Laboratory for Germplasm Innovation & Utilization of Horticultural Crops, College of Horticulture and Forestry Sciences, Huazhong Agricultural University, Wuhan 430070, China; National Key Laboratory for Germplasm Innovation & Utilization of Horticultural Crops, College of Horticulture and Forestry Sciences, Huazhong Agricultural University, Wuhan 430070, China; National Key Laboratory for Germplasm Innovation & Utilization of Horticultural Crops, College of Horticulture and Forestry Sciences, Huazhong Agricultural University, Wuhan 430070, China; Hubei Hongshan Laboratory, Wuhan 430070, China

## Abstract

Vacuole largely dictates the fruit taste and flavor, as most of the sugars and organic acids are stored in the vacuoles of the fruit. However, difficulties associated with vacuole separation severely hinder identification and characterization of vacuolar proteins in fruit species. In this study, we established an effective approach for separating vacuoles and successfully purified vacuolar protein from six types of citrus fruit with varying patterns of sugar and organic acid contents. By using label-free LC–MS/MS proteomic analysis, 1443 core proteins were found to be associated with the essential functions of vacuole in citrus fruit. Correlation analysis of metabolite concentration with proteomic data revealed a transporter system for the accumulation of organic acid and soluble sugars in citrus. Furthermore, we characterized the physiological roles of selected key tonoplast transporters, ABCG15, Dict2.1, TMT2, and STP7 in the accumulation of citric acid and sugars. These findings provide a novel perspective and practical solution for investigating the transporters underlying the formation of citrus taste and flavor.

## Introduction

Citrus is an important fleshy fruit for human health and plays a vital role in agricultural economy and food industry worldwide [1–3]. The most widely known citrus species include mandarin (*Citrus reticulata*), sweet orange (*Citrus sinensis*), pummelo (*Citrus grandis*), grapefruit (*Citrus paradise*), and lemon (*Citrus limon*) [4]. Meanwhile, the facile interspecific hybridization of citrus has resulted in diverse fruit quality attributes, encompassing fruit size, aroma profile, sweetness, acidity, as well as vitamin and flavonoid composition [5, 6]. The combination of these traits collectively determines the citrus flavor and nutritional value, which directly impacts customer acceptance and economic value. Among them, sweetness and acidity, the main components of citrus taste and flavor quality, are primarily determined by the type and concentration of soluble sugar and organic acids [7, 8]. The levels of organic acids and soluble sugars are regulated by metabolism and storage processes [9, 10]. In terms of metabolism, the synthesis and degradation pathways for major soluble sugars and organic acids have been extensively studied [11], and the physiological functions and regulatory modes of key structural genes have also been widely reported [12–14]. However, the specific transporters and mechanisms responsible for the transport and accumulation of these metabolites in citrus remain unknown, despite ample evidence indicating their storage within the central large vacuole [5, 15–17].

Vacuoles exist in almost all vegetative tissues with a variety of physiological functions [18, 19]. The specificity function of vacuole is typically determined by the tissue-specificity in plants. For instance, in orchid flower, the vacuoles accumulate large amounts of carbohydrates or terpenoids for sepal growth or nectar secretion, which facilitates pollen dispersal [20, 21]. In guard cells, vacuoles can control the opening and closing of stomata by regulating turgor pressure [22]. For specialized organs such as fruit or fleshy root, the vacuole provides the main driver for later growth of the yielding organ through expansion [23, 24]. More importantly, it is the primary storage site for metabolites such as sugars [16, 25], organic acids [5], and the others [26]. Because the most basic function of the vacuole is storage, it is crucial to understand how large molecule solutes enter and exit the vacuole, especially for sugar and organic acids that largely determine the taste and flavor of fleshy fruits [27–31]. The transporters or channels localized in the tonoplast are recognized as being responsible for the transportation or exchange of sugar and organic acids between the vacuole and cytosol in fruit or fleshy root cells [18, 23]. For example, the vacuolar channel Aluminum-activated malate transporter 9 (VvALMT9) mediates malate and tartrate accumulation in berries of *Vitis vinifera* [32]. Apple tonoplast-localized malic transporter Ma1 mediates malate storage in the vacuole for controlling fruit acidity [33]. Citrus Cit1, the homolog of the *Arabidopsis* malate transporter tDt, was reported to mediate the citrate export from vacuole [34]. Sugar transporters located on the vacuole membrane have been reported to play a crucial role in maintaining cellular sugar homeostasis and metabolic balance in fruit, such as in apple [31], watermelon [35], and peach [36]. In maize, the vacuolar sucrose transporter ZmSUT2 exports sucrose at night to support plant growth and grain filling [37]. Moreover, sugar accumulation in the vacuole depends on reverse H^+^ transport, while organic acids require protons to establish an acidic vacuolar environment [38]. The proton pumps located in the tonoplast, like H^+^-ATPase/PPase, pump H^+^ into the vacuole to establish a steep proton concentration gradient across the tonoplast, which provides sufficient electrochemical potential gradient for the accumulation of organic acid and soluble sugars [16, 19, 39, 40].

Although sugar/acid transporters have been studied in other species, those in citrus fruit and their regulatory mechanisms remain unclear to date [41]. Compared to a traditional genetic approach, proteomic technology is a more potent tool for large-scale identification of unknown proteins. Up to this point, this technology has been successfully applied to the analysis of novel protein functions in plant stress responses [42, 43], fruit development [44, 45], and fruit quality [46]. However, due to the challenges associated with obtaining sufficient and intact vacuoles, most proteomic studies have focused on the specific tissue or whole-cell level. Ascertaining the relationship between vacuole/tonoplast proteins and metabolite accumulation amidst a macro protein background remains a formidable task. Currently, the proteomic analysis of vacuoles is limited to *Arabidopsis* [47, 48], barley [49], cauliflower buds [50], sugar beet [23], and a very few fruit crops like grape and fig [51, 52]. In citrus, up to now, there is no subcellular organelle (vacuole) level proteomic report to reveal the potential transporters for fruit quality formation.

Citrus fruits have a special juice sac structure [53], and the vacuoles for storing juice can occupy more than 90% of the cell volume, which makes the vacuole very fragile and difficult to separate. Here, we developed a vacuole isolation method for citrus flesh, and used a label-free quantitative proteomics approach to analyse the association between tonoplast transporters and primary soluble sugar and organic acids accumulation.

## Results

### The contents of sugars and acids in citrus fruit development stages

**Figure 1:**
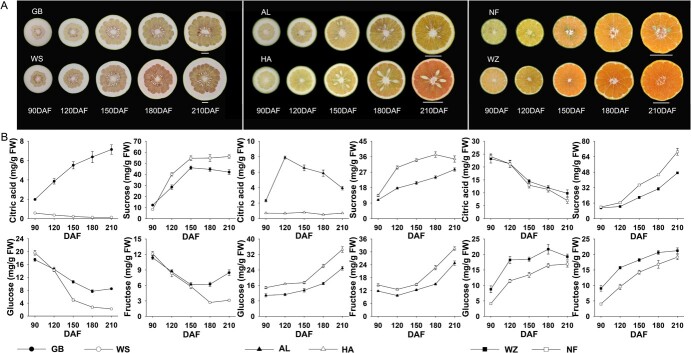
Photos, organic acid and sugar contents of six different citrus during development stages **A** Transverse section characteristics of six different citrus during development. **B** The citric acid, sucrose, fructose, and glucose contents in six citrus genotypes during the whole development stage. Metabolite levels were averaged over two years of 2021 and 2022. Three independent biological replicates were measured for each citrus species. DAF: days after flowering. Bars = 2.5 cm.

The development of citrus fruit can be divided into three stages: cell division (stage I), cell expansion with water accumulation (stage II), and fruit maturation (stage III). At stage II, cessation of cell division triggers transitions in cellular morphology and physiology, leading to rapid fruit enlargement and metabolite accumulation [54]. Here, six different citruses were selected to measure the content of soluble sugars and organic acids, including pomelo (Gao Ban, GB and Wu Suan, WS), sweet orange (An Liu, AL and Hong An, HA), and tangerine (Wen Zhou satsuma citrus, WZ and Nan Feng mandarin, NF) (Fig. 1A). Compared to quinic acid and malic acid, citric acid was the main organic acid, which accumulated rapidly in the early developmental period (120 ± 30 DAF) and maintained quite a high level (4–24 mg/g FW) (Fig. 1B; Fig. S1, see online supplementary material). However, there were significant differences in the selectivity of natural pomelo (*C. grandis*) species WS and GB for citric acid accumulation (Fig. 1B). Meanwhile, HA, a bud mutant of sweet orange (*C. sinensis*), displayed significant alterations in metabolites compared to its wild-type AL [55], including abnormal patterns of citric acid accumulation (Fig. 1A and B). In terms of sugars, sucrose is the primary soluble sugar in pomelo, with a higher concentration in WS compared to GB. The accumulation of sucrose, fructose, and glucose was observed in both sweet oranges and tangerines. However, the concentration of these three sugars was significantly higher in HA compared to AL. Additionally, the hexose concentration in WZ is notably higher than that in NF, while the sucrose level in NF is significantly elevated. These findings show various contents of sugar and acid among citrus varieties (Fig. 1B), implying differential accumulation patterns involved in the process.

### Vacuole isolation and protein purity detection

**Figure 2:**
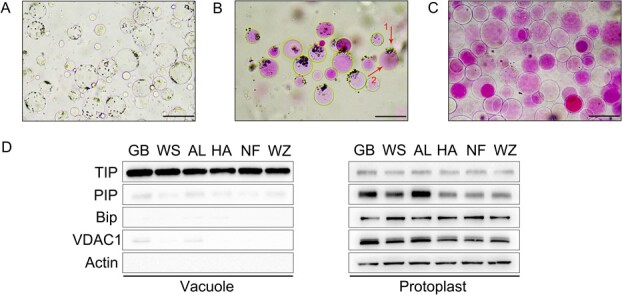
**.** Vacuole isolation and purity evaluation. **A** The isolated protoplasts from citrus fruit juice sacs. **B** Neutral red-stained vacuoles were released from the broken protoplasts in 10% ficoll hypotonic buffer. Arrow 1 indicates protoplast fragments and released organelles, and arrow 2 indicates vacuoles stained with neutral red. **C** Vacuoles were enriched after centrifugation in discontinuous ficoll gradient medium. **D** Western blot analysis of vacuole and protoplast proteins from indicated six citrus genotypes. A total of 10 μg of vacuole protein and protoplast protein were separated by 12% SDS-PAGE and blotted onto PVDF membranes. Blots were performed using different marker antibodies against PIP (protoplast membrane marker), γ-TIP (tonoplast marker), Bip (endoplasmic reticulum marker), and VDAC1–5 (mitochondrial outer membrane marker). Actin protein was used as an internal standard. The vacuoles for each sample were derived from three independent biological replicates. Bars = 100 μm.

Vacuoles function as the main reservoir in fruit, containing a significant amount of sugars, organic acids, and secondary metabolites that largely determine quality of fruit taste and flavor [53]. The stage at 120 DAF was identified as the most representative stage for dynamic changes in citrus sugars and acids content during fruit development (Fig. 1B). Therefore, we selected juice sacs from this period to isolate vacuoles. The protoplasts of the juice sacs were obtained through enzymatic digestion (Fig. 2A). Subsequently, they were incubated in a hypotonic buffer solution to release vacuoles (Fig. 2B). The mixture containing released vacuoles was then centrifuged in Ficoll discontinuous density gradient medium, and intact vacuoles were collected at the interface between 4% Ficoll and vacuole buffer (Fig. S2, see online supplementary material). Under microscopic examination, the purified vacuoles were stained with neutral red and showed no discernible contamination from other organelles or intact protoplasts (Fig. 2C). Subsequently, immunoblot assay was performed to further assess the purity degree of vacuolar proteins via using the specific antibodies for subcellular marker proteins (plasma membrane marker PIP, endoplasmic reticulum marker Bip, mitochondrial outer membrane marker VDAC1–5 and tonoplast marker γ-TIP). As expected, the purified vacuole samples had a strong signal of tonoplast-localized γ-TIP protein, but no obvious or just a much weaker signal detected for PIP, Bip, VDAC1–5, and Actin protein. In contrast, strong signals were detected in the protoplast samples for all four marker proteins, with only γ-TIP being relatively weaker (Fig. 2D). Therefore, it can be concluded that the vacuole samples were highly purified and can be used to perform the vacuolar proteomic analysis.

### Overview of the citrus vacuolar proteome

**Figure 3:**
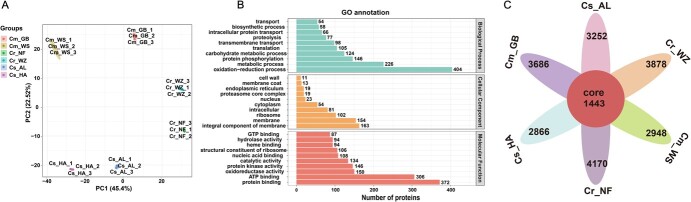
Holistic analysis of the vacuolar proteome and classification of protein functions. **A** Principal component analysis (PCA) of six citrus vacuoles and their independent replicates. The result showed good reproducibility between replicates of each citrus vacuolar proteome. **B** Top 10 GO functional classification of all proteins in six citrus vacuoles. **C** A total of 1443 proteins were shared by six citrus vacuolar proteomes, which were detected simultaneously in at least two biological replicates.

To investigate the potential correlation between vacuole and citrus flavor quality formation, we conducted a label-free LC–MS/MS proteomic analysis on vacuole components from six citrus samples to identify vacuolar proteins, yielding a total of 36 928 credible spectrum peptides. To achieve optimal coverage, a combination of three protein databases for pomelo, sweet oranges, and tangerine was utilized in protein searches. A total of 4931 non-redundant proteins were identified from six samples based on the screening criterion of being detected in at least one biological replicate (Table S1). From this, 3356, 2556, 2827, 2450, 3856, and 3557 vacuolar proteins were identified in six samples of GB, WS, AL, HA, NF, and WZ, respectively (Fig. S3A, see online supplementary material). Among these 4931 proteins, most of their molecular weights are in the range of 10–70 kDa (Fig. S3B, see online supplementary material), which is consistent with previous report in *Arabidopsis* tissue [47]. Principal component analysis (PCA) showed that the proteomes of three replicates from each citrus sample clustered together and exhibited good repeatability (Fig. 3A). GO (Gene Ontology), COG (Cluster of Orthologous Group), KEGG (Kyoto Encyclopedia of Genes and Genomes), and IPR (InterPro) databases were used for functional annotation of these 4931 proteins (Fig. S4, Table S1, see online supplementary material). In the results of GO annotation, 36.8% (1339) proteins are involved in material and energy metabolic processes, 21.9% (798) of the proteins have kinase and catalytic activity, 17.6% (640) of the proteins act as components of biological membranes or are involved in transmembrane transport process. In addition, 5.2% (190) of the proteins function in protein degradation (Fig. 3B; Table S2, see online supplementary material). These functions or biological processes are closely associated with the storage of metabolites or ions that accompany energy expenditure. Moreover, 21.9% (798) of the proteins exhibited kinase or phosphorylation activity, implying that protein-level modifications regulate certain protein functions in the vacuole.

Among the proteins detected in at least two biological replicates, a total of 1443 members shared by six citrus samples were considered as core proteins that potentially represent the functions of citrus vacuoles (Fig. 3C; Table S3, see online supplementary material). GO enrichment analysis reveals that their functions are mainly involved in protein glycosylation modification and degradation, material and energy metabolism, ATP-dependent transmembrane transport, defense and stress response (Fig. S5, see online supplementary material). These functions are essential for vacuoles involved in fruit rapid development and metabolite accumulation.

### Identification of tonoplast transporters and channels from citrus vacuolar proteome

**Figure 4:**
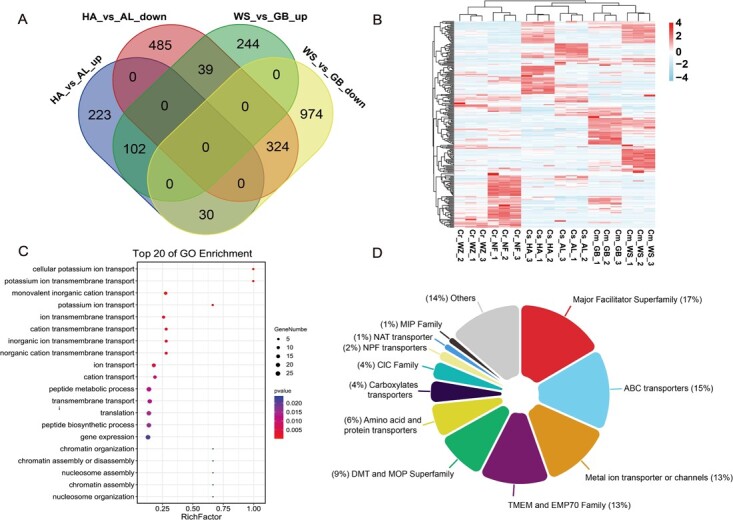
Quantitative and differential protein analysis of the vacuolar proteome **A** Differential protein analysis within the vacuole proteome of sweet orange (HA, AL) and pomelo (WS, GB). **B** Analysis of the expression patterns of 426 DAPs in six citrus vacuoles. The cultivars with similar acidity (WZ vs NF) have similar expression patterns, and the cultivars with disparity in acidity (HA vs AL, WS vs GB) have distinctly different expression patterns. **C** The GO functional enrichment of the co-upregulated and co-downregulated 426 DAPs in the two groups of citrus with significant differences in citric acid content, which were significantly enriched in the biological process of transmembrane transport. **D** All 164 transporters were classified into 12 categories based on functional annotation and structure analysis.

Differentially abundant proteins (DAPs) are frequently linked to variations in traits. A quantitative analysis was performed utilizing an intensity-based absolute quantification (iBAQ) protocol to further evaluate the variability of vacuolar proteomes among the three citrus groups. Interestingly, samples (NF vs WZ) with similar citric acid content (23 ± 2 mg/g FW) exhibited a more balanced number of up-regulated and down-regulated proteins, whereas those with disparate differences in acidity (HA vs AL and WS vs GB) showed a greater proportion of down-regulated expressed proteins compared to up-regulated ones (Fig. S6, Tables S4–S6, see online supplementary material). There were 102 co-up-regulated and 324 co-down-regulated proteins in the low-acid citrus (WS and HA) compared to the high-acid citrus (GB and AL) (Fig. 4A). Corresponding to fruit acidity, the expression patterns of these 426 proteins varied significantly among the sweet orange (HA and AL) and pomelo group (WS and GB), while exhibiting relative similarity within the tangerine group (WZ and NF) (Fig. 4B). The GO enrichment analysis revealed that the 426 DAPs were significantly enriched in transmembrane transport-related pathways (Fig. 4C), indicating that tonoplast-localized transporters play a crucial role in metabolite accumulation within vacuoles. Therefore, we conducted a thorough screening of vacuolar proteome data to identify potential transporters.

The transporters were identified from all vacuolar proteins according to the following filtering scheme. First, members containing transmembrane domains (TMD) were filtered out, yielding a total of 1257 proteins. Followed by those members containing only signal peptide were subsequently discarded, as signal peptides would also default to the transmembrane domain, resulting in 1085 membrane proteins for further analysis (Table S7, see online supplementary material). Subsequently, these 1085 proteins were matched in the transporter database (TCDB), combined with GO annotation, and eventually 164 members were designated as transporters (Fig. 4D, Table S8, see online supplementary material). Among them, 25 are proton pumps including 18 P-type ATPase (P-ATPase) superfamily members, four pyrophosphatase (V-PPase) proton pump family members and 3 V-type proton ATPase (V-ATPase), 23 are major facilitator superfamily (MFS) proteins, and 21 are ATP-binding cassette superfamily members. In addition, 18 metal ion transporter or channels, 18 transmembrane protein (TMEM) and the endomembrane protein-70 (EMP70) members were also identified. The drug/metabolite transporter (DMT) superfamily and the multidrug/oligosaccharidyl-lipid/polysaccharide (MOP) flippase superfamily had totally 13 members in here. There are also some categories with fewer members, such as amino acid or protein transporters (nine), carboxylate transporters (six), chloride ion-related transporters (five), NRT1/PTR family transporters (NPF) (three), the major intrinsic proteins (MIP) (two), and vitamin C transporters (two). Finally, there remain 19 transporters whose functions have yet to be assigned. Overall, the citrus vacuolar proteome data has revealed a diverse and novel array of tonoplast-localized transporters, indicating that further exploration into the function of the citrus vacuole is warranted.

### Key transporter or channels for vacuolar accumulation of sugar or acids

**Figure 5:**
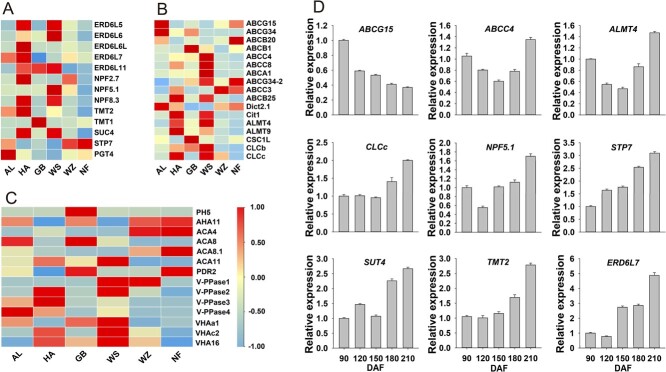
Protein abundance and developmental transcript levels of sugar or acid-related transporter or channels. **A** Abundance of transporters associated with sugar accumulation in the vacuolar proteome of six different citrus. **B** Abundance of transporters or channels associated with organic acids accumulation in the vacuolar proteome of six different citrus. **C** Abundance of proton pumps associated with accumulation of organic acids or soluble sugars in the vacuolar proteome of different citrus. **D** Transcription levels of selected transporter coding genes in sweet orange fruit development stage. The values represent the means ± SD of three biological replicates.

Close correlation between tonoplast sugar transporters and fruit sugar accumulation have been documented in apple or other fruit crops [31, 56–58]. Nevertheless, tonoplast sugar transporters for sugar accumulation in citrus fruits remains elusive. The sugar levels of the sweet orange and tangerine fruits utilized in this study exhibited significant differences (Fig. 1B; Fig. S1, see online supplementary material). By integrating protein expression levels and protein function annotation, we identified 13 potential sugar transporters, including Early-Response to Dehydration six-Like proteins (ERD6Ls), NPF-like proteins, Tonoplastic Monosaccharide Transporters (TMTs), Plastidic Glucose Transporters (PGTs), Sucrose Transporters (SUTs) and Sugar Transporter Proteins (STPs). Among them, the protein abundance of ERD6L-5/6/7, NPF5.1, NPF8.3 as well as TMT2 and SUT4 was highly consistent with the difference in sugar content between sweet orange or tangerine groups (Fig. 5A). Moreover, their mRNA expression levels gradually increased during fruit development in parallel with the trend of sugar accumulation in sweet orange (Fig. 5D). For instance, the hexose concentration in WZ exhibited a significantly higher level than that in NF (Fig. 1B). Correspondingly, the protein abundance of TMT2 and NPF8.3 in WZ was also found to be 3-fold and 2-fold higher than that observed in NF (Table S8, see online supplementary material). It is worth noting that an NPF-like protein SUGCAR1 was recently reported to have sugar transport activity [59]. However, its involvement in fruit sugar accumulation remains unexplored, highlighting its potential as a novel regulator of fruit sweetness quality.

The transport process significantly affects the accumulation level of citric acid, which is the primary organic acid in citrus fruits. Here, a total of 17 potential transporter or channels, including ATP-binding cassette (ABC) transporters, carboxylic acid transporters and 2 chloride channels (CLCs) were screened through correlation analysis between protein abundance and citric acid concentration to identify their possible involvement in citrate accumulation (Fig. 5B). It has been reported that Cit1 possesses the capability of exporting citric acid from the vacuole [34], which is in line with the vacuolar proteome data indicating a higher protein level of Cit1 in low-acid citrus such as HA and WS (Fig. 5D). Dicarboxylate transporter 2.1 (Dict2.1) is a newly annotated dicarboxylate transporter, and its protein level in AL is about 1.6-fold higher than that in HA. ABCG15 exhibited high protein level in AL and GB with elevated citric acid concentration, whereas its expression was negligible in acid-free citrus HA or WS (Fig. 5B; Table S8, see online supplementary material). Additionally, the transcript levels of *ABCG15* in sweet orange was consistent with the pattern of citric acid accumulation during development (Fig. 5D), suggesting their active involvement in this process. In contrast, the protein abundance and developmental transcript levels of *ABCC4*, *ALMT4*, and *CLCc* in the pomelo or sweet orange group exhibit an opposite trend with citric acid content (Fig. 5B and D). Among them, the abundance of ABCC4 in HA and WS was respectively 2-fold and 3-fold higher than that in AL and GB (Table S8, see online supplementary material), suggesting that they may be involved in citric acid export or hinder its accumulation in the vacuoles.

Proton pumps transport H^+^ protons into the vacuole, creating an electrochemical potential gradient that drives proton-coupled sugar transport and serves as an ‘acid trap’ for organic acids accumulation [38]. It is well-established that PH5, a P_3A_-type proton pump, played a significant role in vacuolar acidification and citric acid accumulation in citrus [5, 60], which was confirmed in our proteomic data that PH5 had a dramatically high protein level in GB than WS fruit [5, 60]. However, it remains unclear whether other new proton pumps participate in the accumulation of citric acid in citrus. Based on correlation analysis between citric acid concentration and protein expression levels, the result suggested that nine other proton pumps may contribute to the accumulation of citric acid, including both P-type and V-type ATPase (Fig. 5C). Among them, the protein abundance of AHA11, ACA8, and VHAa1 in high acidity AL were respectively found to be 10-fold, 3-fold, and 3-fold higher than that in low acidity HA **(**Fig. 5C; Table S8, see online supplementary material**)**, indicating a positive effect on citric acid accumulation. The V-PPase is commonly associated with sugar accumulation [16], and we identified four members of the V-PPase family in this study. The protein level of V-PPase1–3 consistently exhibited higher expression in high-sugar varieties (Fig. 5C; Table S8, see online supplementary material), which aligns their crucial functions in sugar accumulation. Therefore, the potential role in sugar/organic acid accumulation of target proton pumps deserves to be further investigated.

### Localization and functional analysis of putative transporters or channel involved in sugar or acid accumulation

**Figure 6:**
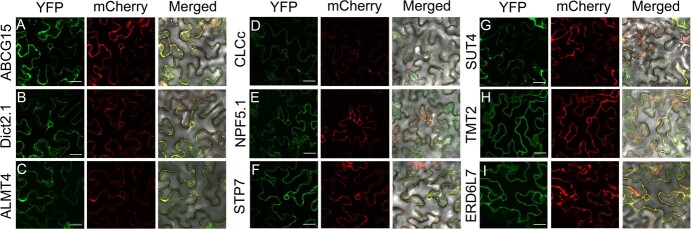
Subcellular localization analysis of nine potential sugar and acid transporters or channel. **A**–**I** Nine proteins were fused in frame with the YFP fluorescence tag and transiently co-expressed with the tonoplast mCherry-marker (AtTIP) in tobacco leaves. ABCG15 (**A**) and Dict2.1 (**B**) are potential transporter proteins responsible for citric acid accumulation. ALMT4 (**C**) and CLCc (**D**) were negatively associated with citric acid accumulation. NPF5.1 (**E**), STP7 (**F**), SUT4 (**G**), TMT2 (**H**), and ERD6L7 (**I**) are transporters that closely related to sugar accumulation. Scale bars = 25 μm.

**Figure 7:**
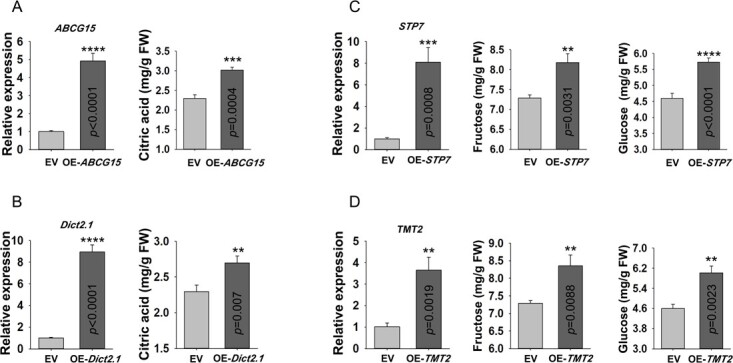
The functional analysis of candidate tonoplast transporters via the transient overexpression in citrus juice sacs. **A** Expression level of *ABCG15* in transformed juice sacs and its effect on citric acid content. **B** Expression level of *Dict2.1* in transformed juice sacs and its effect on citric acid content. **C** Expression level of *STP7* in transformed juice sacs and its effect on fructose and glucose content. **D** Expression level of *TMT2* in transformed juice sacs and its effect on fructose and glucose content. Error bars represent the SD of three independent biological replicates. SPSS, version 22, was used to analyse the variation between the data (^****^P<0.0001, ^***^P < 0.001, ^**^P < 0.01) .

To verify whether the screened potential sugar/acid-related transporters or channel were truly localized in the vesicular membrane, the protein-YFP signals were observed with the tonoplast marker AtTIP-mCherry, including citric acid accumulation-related transporters or channel ABCG15, Dict2.1, ALMT4, CLCc, and sugar accumulation-related transporters NPF5.1, SUT4, STP7, TMT2, ERD6L7. Compared with the empty vector (Fig. S7), all nine members produced a circular fluorescent signal that wrapped around the nucleus and co-localized with the tonoplast mCherry marker (Fig. 6; Fig. S8, see online supplementary material), demonstrating that they were localized to the vacuole membrane. This validates the reliability of the vacuolar proteomic data in this study and supports the speculation that these members may contribute to vacuolar accumulation of citric acid or soluble sugars. The transient transformation assay of citrus juice sacs, involving *ABCG15*, *Dict2.1*, *STP7*, and *TMT2*, was conducted to further test the function of target transporters. As shown in Fig. 7, overexpression of *ABCG15* and *Dict2.1* resulted in significant increase in citric acid concentration within citrus juice cells; overexpression of *STP7* and *TMT2* led to higher levels of fructose and glucose. The findings suggest that the vacuolar proteome serves as a valuable foundation for the identification of novel tonoplast proteins involved in sugar and acid accumulation, thereby enhancing fruit taste and flavor quality.

## Discussion

For fruit crops, efficient isolation of vacuoles has been a challenging aspect of their functional research. In this study, we successfully isolated and enriched citrus flesh vacuoles for the first time. By comparative proteomics analysis, we identified tonoplast transporters and investigated their roles in the accumulation of citric acid and sugars in citrus. This approach provides a new way to understand the regulatory pathways underlying citrus taste and flavor quality.

Identification and analysis of vacuolar proteins is crucial for comprehending the role of vacuoles, yet only one report each on grapes [52] and figs [51] is available for vacuolar proteomic analysis among fruit crops. The unique characteristics of citrus juice sacs present a significant challenge in the isolation of vacuoles. To overcome this, we developed the following steps to obtain sufficient intact vacuoles. First, it is essential to ensure sufficient cleavage of the juice sacs to induce complete exposure of the cells to enzymatic digestion, which facilitates obtaining a higher yield of protoplasts (Fig. 2A; Fig. S2; see online supplementary material). Second, higher concentrations of cellulase (2%) and macerozyme enzymes (1%) can significantly reduce the enzymatic digestion time, thereby ensuring maximum protoplast freshness. Subsequently, the vacuole was released from the protoplasts by absorbing water and rupturing in a buffer solution with an appropriately low osmolarity (Fig. 2B). In addition, low temperatures during this process are necessary to maintain vacuole integrity. Finally, optimal centrifugation condition (2600 × *g* for 15 min) facilitates the aggregation of individual vesicles in medium with varying density gradients (Fig. S2, see online supplementary material). It is worth noting that the vacuoles in citrus flesh appear to be larger than those in *Arabidopsis* [47], beet root [23], or fig [51], which is consistent with the juicy nature of citrus flesh (Fig. 2C). By using this protocol, we isolated flesh vacuoles from three type of citrus, tangerine, sweet orange, and pomelo, indicating the suitability of this scheme for most types of citrus.

The assurance of vacuole sample purity is essential for facilitating subsequent analysis. However, complete removal of the small amount of plasma membrane attached to the vacuole was not possible due to interdependence among organelles. The presence of a weak PIP signal suggests that trace amounts of plasma membrane fragments still remain in the vacuolar preparations (Fig. 2D), which aligns with findings from previous studies [61]. Finally, a total of 4931 non-redundant proteins were identified in vacuole samples from six different citrus samples, which greatly surpasses the number of proteins that can be obtained using vacuolar membranes as materials [47, 50]. This comprehensive identification facilitates a deeper understanding of citrus vacuole functions such as energy metabolism, transmembrane transport and protein modifications. Among them, energy metabolism serves as the foundation of vital activities and a prerequisite for the transmembrane transport of organic substances. Meanwhile, transporters shuttle metabolites across membranes, which are regulated by modifications such as phosphorylation. That is why some phosphorylated proteins also have been detected in vacuole proteome data. Additionally, during development, fruit cells transition from increasing numbers to expanding size with a concomitant change in the expression pattern of associated proteins, which leads to the degradation of cytokinesis-associated proteins and the generation of new proteins, as observed in apple [45], grape [52], fig [51], and decalepis [62].

Given the crucial storage function of the vacuole and the pivotal role of vacuolar membrane proteins in metabolite transport and accumulation [63], we conducted a protein abundance and metabolite content correlation analysis to specifically identify tonoplast transporters related to citrus taste and flavor. These included sugar transporters, acid accumulation-related transporters, and proton pumps. Here, ERD6Ls, NPF-like proteins, TMT2, SUT4, and STP7 were suggested to be closely associated with soluble sugar accumulation. The abundance of both ERD6Ls and TMT2 was significantly higher in high-sugar types (HA, WZ) compared to low-sugar types (AL, NF) (Figs 1Band 5A), which is similar as the research work in apple [31, 64]. Furthermore, we also identified a STP7 protein that is localized to the tonoplast (Fig. 6), whereas its family members were previously reported to be exclusively located at the plasma membrane [65, 66]. The transient overexpression analysis demonstrated a positive correlation between STP7 and hexose content in juice sacs (Fig. 7), providing the foundation for further investigation into the new role of STP members in sugar accumulation in the vacuole. The NRT1/PTR Family (NPF) proteins are typically responsible for the transport of nitrates, peptides, and ions. However, recent research has revealed that maize NPF member SUGCAR1 functions as a novel transporter of sucrose/glucose during the grain filling stage in maize [59]. Accordingly, we hypothesize that NPF-like proteins within the vacuolar proteome may serve as new members implicated in vacuolar sugar accumulation, given its localization to the vacuolar membrane and the results of our correlation analysis on sugar level (Figs 5 and6).

For the citric acid transporter, Cit1 was previously reported to be responsible for exporting citric acid from the vacuole to the cytoplasm [34]. From this perspective, the high level of Cit1 serves as a significant contributing factor to the remarkably low citric acid level detected in WS and HA (Fig. 5B). Besides, studies have demonstrated that the transport of citrate and malate into and out of the vacuole is facilitated by either a single transporter or two highly similar transporters, suggesting a shared mechanism for their transportation [67–69]. Increasing evidence has demonstrated that ALMTs function as malate transporters into the vacuole, exemplified by Ma1 (MdALMT9) in apples which confers fruit acidity through importing malate into the vacuole [33]. In our findings, the protein levels of tonoplast ALMT4/9 were significantly elevated in low-acid citrus (WS) compared to high-acid citrus (GB) (Figs 1B and5B), and this is probably why WS has much higher malic acid content than GB (Fig. 1B; Fig. S1, see online supplementary material). Furthermore, the transcript of ALMT4 during development exhibits an inverse correlation with citric acid level (Fig. 5B), implying a potential role of ALMT4 in citric acid efflux from citrus vacuole. In addition, the vacuolar proteome data also revealed the presence of members belonging to the ATP-binding cassette (ABC) superfamily. Plant ABC transporters exhibit a wide range of substrates encompassing diverse metabolites, hormones, lipids, and metal ions; however, their specific physiological functions remain largely unexplored [70]. Lee *et al.* discovered that AtABCB14 facilitates the import of malate from the apoplast into guard cells and regulates stomatal response to CO_2_, indicating that ABC transporters have the structural basis for transporting carboxylate [71]. Our findings support that ABCG15 may play a positive role in citric acid accumulation in citrus, as evidenced by both its protein abundance and transcript levels during fruit development (Fig. 5). Subcellular localization analysis and juice sacs transfection results further confirmed that the tonoplast localized ABCG15 significantly enhanced citric acid content in juice sacs (Figs 6 and7).

The Type I V-PPase is primarily located in the tonoplast, and its role involves hydrolyzing PPi to generate energy for transmembrane transport while also contributing to the storage of photosynthetic assimilates within the vacuole [16, 72, 73]. In this work, three new members of V-PPase, namely V-PPase1–3, were found to have higher abundance in high-sugar citrus than in low-sugar citrus (Table S8, see online supplementary material), which further supports the previous investigation that type I V-PPase members CsVPP-1 and CsVPP-2 play key roles in sucrose storage in the vacuole [16]. Moreover, citrus vacuoles can achieve remarkably low pH levels, reaching as low as 2 in some fruits [74]. It is possible that a P-type ATPase with greater acidification ability is responsible for the higher acidity of citrus vacuoles. Previous studies have shown that CsPH5 (AHA10), a P_3A_-type ATPase in citrus, plays a positive role in organic acid accumulation [5], which is confirmed in our results in relation to protein and acid content (Fig. 5C). Moreover, as AHA11 and ACA8 also showed a trend consistent with CsPH5, it is likely that both affect citric acid accumulation in a similar manner as PH5 proton pump (Fig. 5C).

**Figure 8:**
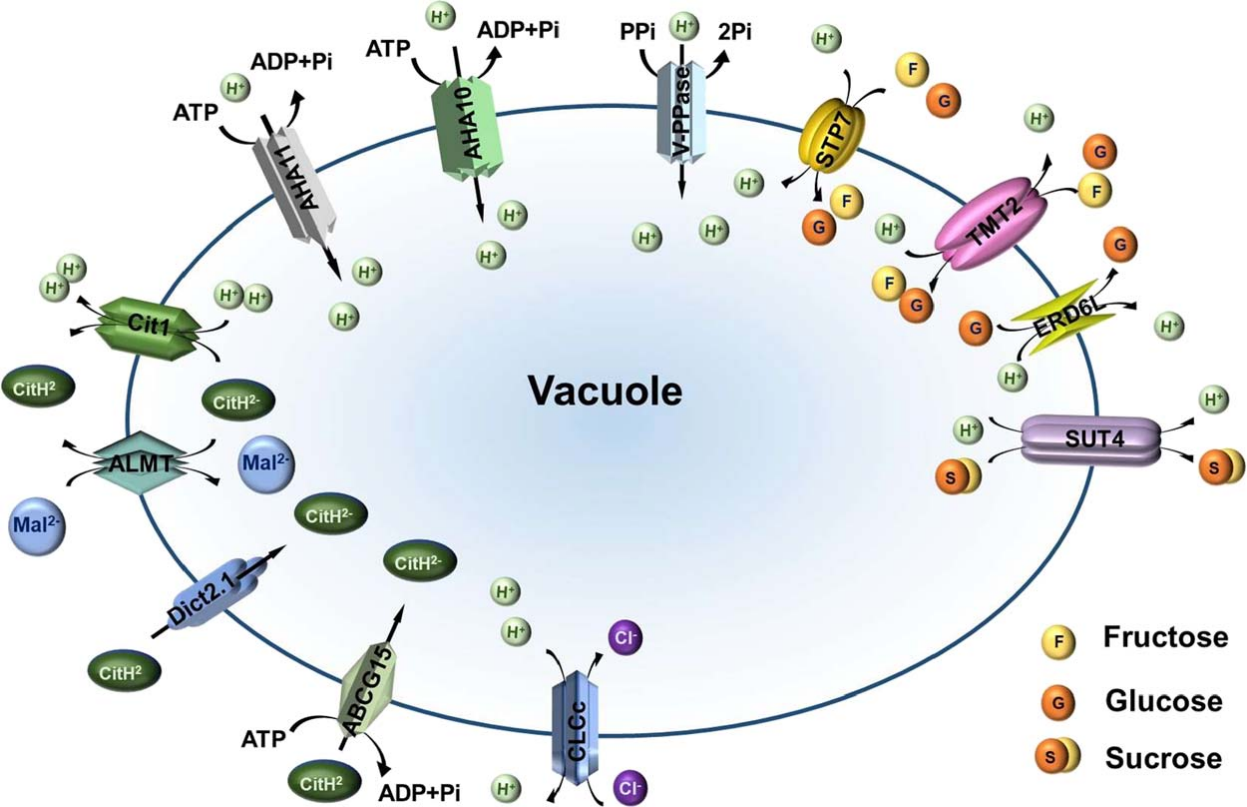
Model of identified vacuolar transports in regulating quality formation in citrus.

Taken together, this study successfully established a citrus vacuole isolation system, which provides the possibility for in-depth investigation of vacuole function. More importantly, the subcellular organelle-level proteome analysis has shed light on the physiological functions of citrus vacuoles and the transport system involved in determining the taste and flavor of citrus fruit. These findings provide valuable insights into understanding the growth and development of citrus fruits as well as quality formation. Finally, the association analysis of sugar and acid levels in different citrus with the proteome has identified key transporters that play a crucial role in the accumulation of sugars and organic acids (Fig. 8). This lays the foundation for further functional characterization of these transporters to gain a comprehensive picture of the molecular basis for taste and flavor quality of citrus and other fleshy fruits.

## Materials and methods

### Plant materials and growth conditions

Mature and healthy citrus trees used in this study were cultivated and managed uniformly at the Citrus Germplasm Resource Center of Huazhong Agricultural University (Wuhan, Hubei, China) under standardized conditions. Fruit samples of ‘Gaoban’ pumelo (GB*, C. grandis* cv. Gaoban), ‘Wusuan’ pumelo (WS*, C. grandis c*v. Wusuan), ‘Anliu’ orange (AL, *C. sinensis* cv. Anliu), ‘Honganliu’ (HA, *C. sinensis* cv. Honganliu), ‘Nanfeng’ tangerine (NF, *C. reticulata* cv. Nanfeng), and ‘Wenzhou’ (WZ *C. unshiu* Marc. cv*.* Guoqing No.1) were harvested at 90, 120, 150, 180, 210 DAF. AL orange fruits were used to detect transcript levels of developmentally relevant proteins. Nine healthy fruits of uniform size and maturity status were selected from one tree as one independent biological replicate, samples of each developmental period contain three biological replicates. Juice sacs were rapidly collected for metabolite content detection and vacuole isolation. All fresh materials were snapped frozen in liquid nitrogen and stored at −80°C until analysis. Tobacco plants (*Nicotiana benthamiana*) were grown in soil pots under long-day photoperiod condition (a light/dark cycle of 16 h/8 h) in a growth chamber at 24°C. HB pumelo fruit (HB, *C. grandis* Osbeck ‘Hirado Buntan’) were harvested at 200 DAF and their fresh juice sacs were used for transient transfection.

### Sugars and organic acids content assay

Sugars and organic acids were extracted in 75% (v/v) methanol as previously described [75]. The ribitol (0.12 mg per sample) was added as an internal standard. One hundred microliters of each aqueous phase sample was dried under vacuum without heat, and then derivatized with methoxyamine hydrochloride and N-methyl-N-trimethylsilyl-trifluoroacetamide sequentially. The Agilent 6890B Gas Chromatography System (Agilent Technologies, Palo Alto, CA, USA) equipped with a flame ionization detector and a non-polar HP-5 (5%-phenyl)-methylpolysiloxane column (30.0 m × 0.32 mm × 0.25 μm) was used to analyse the sugars and organic acids content. Three independent extractions were performed for per sample.

### Vacuole isolation

For vacuole isolation from citrus fruit, 20 g of chopped fruit pulp was gently shaken (60–80 rpm) in 0.7 M mannitol solution for 20 min, followed by a mild incubation in washing buffer (0.7 mannitol, 10 mM MES, 1 mM CaCl_2_, pH 5.8) containing 2% cellulase R-10 (Yakult Honsha, Tokyo, Japan), 1% Macerozyme R-10 (Yakult Honsha, Tokyo, Japan) and 0.1% BSA for 2 h at room temperature for the digestion of flesh. The protoplasts were filtered with a 75 μm pore size mesh and collected by centrifugation at 600 rpm for 5 min, washed twice with pre-cooled (4°C) washing buffer. Vacuoles were released from protoplasts in lysis buffer containing 0.2 M mannitol, 10% (w/v) Ficoll, 15 mM EDTA, 2 mM DTT, 10 mM HEPES, 0.005‰ Neutral red, pH 8.0. The vacuoles were subsequently collected via the discontinuous density gradient centrifugation. The 4% Ficoll intermediate layer was prepared in proportion to 10% Ficoll lysis buffer and vacuole buffer containing 0.5 M mannitol and 10 mM HEPES (pH 7.5). After centrifugation at 2600 × *g* for 20 min at 4°C, the rose-red vacuoles between the middle and top layers were carefully collected.

### Total vacuolar proteins extraction and western blot analysis

The total vacuolar protein was extracted by the general sample preparation method for proteomics assay [76–78]. After ultrafiltration, the vacuolar samples were dissolved in dissolution buffer containing 8 M urea, 100 mM TEAB (pH = 8.5). Subsequently, 10 mM DTT was added to react at 56°C for 1 h. Sufficient IAM (iodoacetamide) was added to react at room temperature for 1 h in the dark. Follow the manufacturer’s instruction, protein concentrations were determined by using a bicinchoninic acid protein assay kit (Beyotime, Shanghai, China). Western Blot was applied to evaluate the purity of vacuolar proteins as described previously [49, 50, 79]. Briefly, 10 μg of protoplasts and vacuolar proteins were separated by 12% SDS-PAGE gel and transferred to PVDF membranes, and then incubated with the following antibodies: tonoplast intrinistic protein 1–1, 1–2 (gamma) (TIP1, tonoplast marker, 23 kDa, AS09 493), plasma membrane intrinistic protein1s (PIP1s, plasma membrane marker, 28 kDa, AS09 489), lumenal-binding protein (BiP, endoplasmic reticulum marker, 80 kDa, AS09 481), and voltage-dependent anion-selective channel protein 1–5 (VDAC1–5, mitochondrial outer membrane marker, 29 kDa, AS07 212). Following incubation in a 20 000-fold diluted secondary antibody (anti-rabbit, coupled to horseradish peroxidase), HRP activity was detected through a chemiluminescent blotting substrate kit (Beyotime Biotechnology, Shanghai, China) according to the manufacturer’s protocol.

### Proteolysis and LC–MS/MS assay

EASY-nLCTM 1200 nanoliter level UHPLC system (Thermo Fisher Scientific, Waltham, Massachusetts, USA) was used for the separation of digested peptides. The peptide mixtures were desalted in solution A containing 0.1% formic acid (v/v) loaded onto a special customized C18 nanoflow trap column (4.5 cm × 75 μm, 3 μm) and subsequently passed through a customized C18 column (15 cm × 150 μm, 1.9 μm) in 4–80% acetonitrile (0.1% formic acid) at a flow rate of 600 nL/min for 70 min gradient separation. The separated peptides were analysed by Q Exactive™ HF-X mass spectrometer (Thermo Fisher Scientific, USA), with ion source of Nanospray Flex™(ESI), spray voltage of 2.1 kV and ion transport capillary temperature of 320°C. Full scan range from m/z 350 to 1500 with resolution of 60 000 (at m/z 200), an automatic gain control (AGC) target value was 3 × 10 [6] and a maximum ion injection time was 20 ms. The parent ions with ionic strength TOP 40 in the scan were detected by secondary mass spectrometry (MS) using higher energy collisional dissociation (HCD), and the normalized collision energy was set as 27% to generate the raw data for MS detection.

### Database search and protein identification

The raw proteome data was analysed using Proteome Discoverer (version 2.2) for peptide identification and protein quantification. The original data was searched against the non-redundant protein databases of *C. sinensis* (version 2.0), *C. grandis* and *Citrus reticulate* (http://citrus.hzau.edu.cn/download.php). The main parameters of Proteome Discoverer used in this study were listed in Table S10 (see online supplementary material). The proteins identified in at least two samples were retained for further analysis. Differentially abundant proteins (DAPs) between two groups were screened with arbitrary fold change >2 and *P* value <0.05 (*t*-test). Protein function annotation was searched against UniProt and EggNOG-mapper databases using diamond (version 0.9.24). Transporters were identified using the Transporter Classification Database (TCDB; http://www.tcdb.org/).

### Bioinformatic analyses

GO and KEGG annotation were performed by EggNOG-mapper with its related database. GO and KEGG enrichment were conducted by agriGO v2.0 database [80] and KOBAS software [81], respectively. The protein subcellular localization was predicted using Cell-PLoc 1.0 package [82]. Venn diagrams were performed by Evenn tool with default parameters [83]. The heatmap of protein expression profile was draw by heatmap package in R version 4.2.0 (https://www.r-project.org/). DAPs between the two proteomes were screened with the abundance of fold change >2 and *P* value <0.05 using Student’s *t*-test.

### Subcellular localization assay

The CDS (coding sequence) sequence of the target protein (without stop codon) was cloned into the pRI101-YFP vector containing the YFP tag and transformed into *Agrobacterium tumefaciens* GV3101 as described previously [84, 85]. AtTIP fused to mCherry was used for co-localization analysis. The recombinant vector was transformed into tobacco leaves by *Agrobacterium* injection, and the fusion protein was expressed under the drive of the CaMV 35S promoter. YFP and mCherry signals were captured in multi-channel mode using a laser scanning confocal microscope (Leica TCS-SP8, Germany). YFP and Deep Red were excited at 514 and 587 nm, and their emission signals were detected at 520 to 540 nm and 600 to 620 nm, respectively. Primers used for gene cloning were listed in Table S9 (see online supplementary material).

### RNA extraction and qRT-PCR

Total RNA was extracted using RNAiso Plus kit (TAKARA, Japan) and potential DNA contamination was removed using DNAase I, and cDNA was synthesized using the Revert Aid™ First Strand cDNA Synthesis Kit (TransGen, Beijing, China) following the manufacturer’s instructions. qRT-PCR was conducted with an QuantStudio™ 7 Flex Real-Time PCR system (Applied Biosystems, Singapore). Each reaction was repeated three times per biological replicate with *CsActin* as internal reference. The primers were listed in Table S8 (see online supplementary material). Data was analysed using the 2^−ΔΔCt^ method [84].

### Transient expression in citrus juice sacs

Follow the previous method with some modifications [86]. The CDS sequence of the target protein (without stop codon) was cloned into 35 s-driven pK7WG2D vector and transformed into *A. tumefaciens* EHA105. Briefly, healthy juice sacs were cut off at one terminal and subsequently submerged in *A. tumefaciens* infestation solution containing 100 μM ACE (Acetosyringone). Suck the liquid on the surface of the juice sacs after vacuum treatment for 20 min. Then the juice sacs were placed on the MS medium containing 0.5 mg/L vitamin C and incubated in the dark for 7 d. The samples were snap-frozen in liquid nitrogen and stored at −80°C until analysis. Primers used for gene cloning are listed in Table S8 (see online supplementary material).

## Supplementary Material

Web_Material_uhad249

## Data Availability

All data is available within manuscript and its supporting materials.
