# Supplementary material for: Vacuolar proteomic analysis reveals tonoplast transporters for accumulation of citric acid and sugar in citrus fruit
Source: Hortic Res. 2023 Nov 28;11(1):uhad249. doi: 10.1093/hr/uhad249 (PMC10822839; doi:10.1093/hr/uhad249)
Supplement: Web_Material_uhad249 [file web_material_uhad249.zip › 20231021_Supplemental Figures_revised.docx]

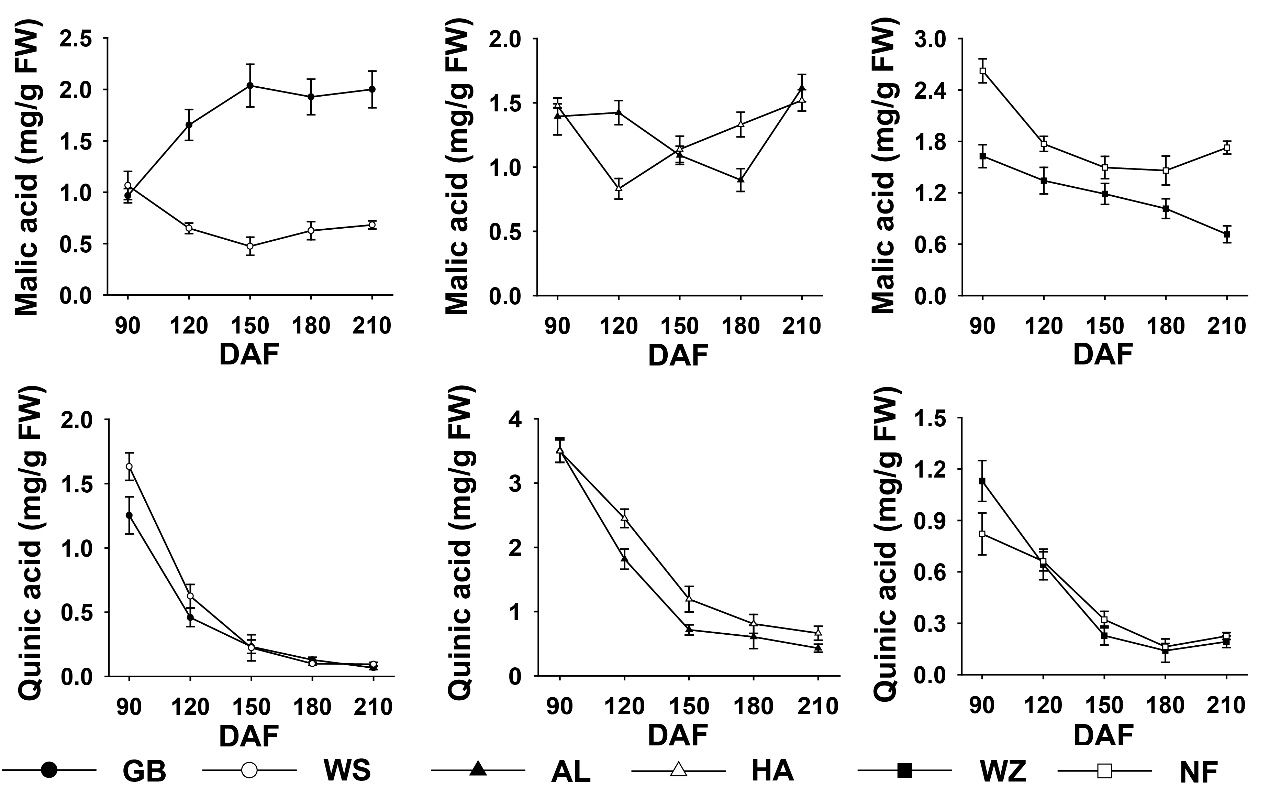


**Figure S1** **Malic acid and quinic acid contents of 6 different citruses during the developmental period.** Three independent biological replicates were measured for each sample. DAF: Days After Flowering. Bar = 2.5 cm.


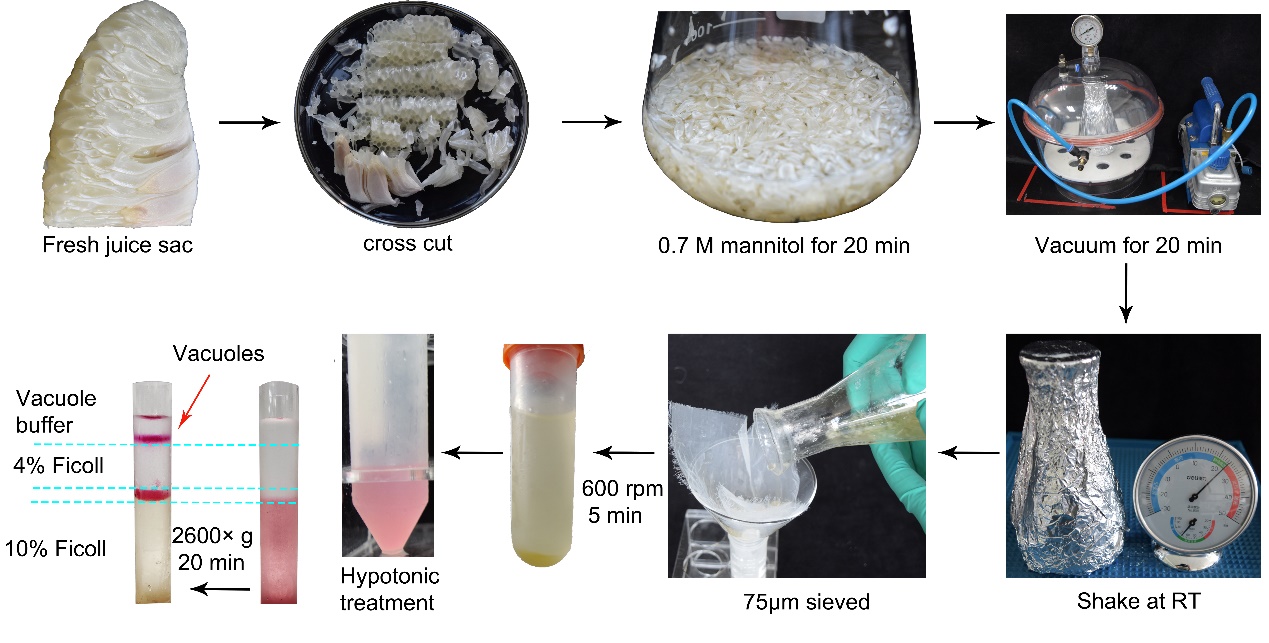


**Figure S2** **Workflow diagram for the isolation and purification of vacuoles from citrus pulp.**


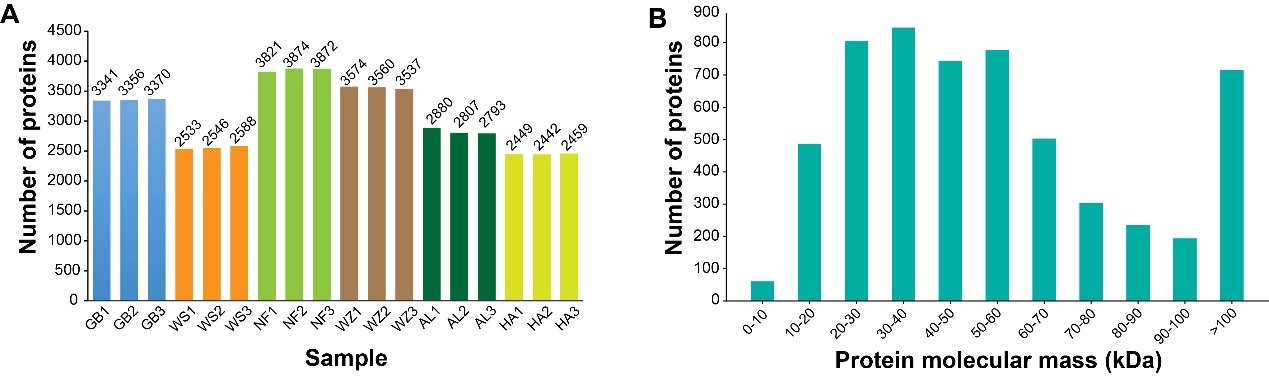


**Figure S3 The number of proteins detected in 6 different citruses and the protein molecular weight.**

A, The number of proteins detected in each citrus sample.

B, Number of proteins in different molecular weight intervals.


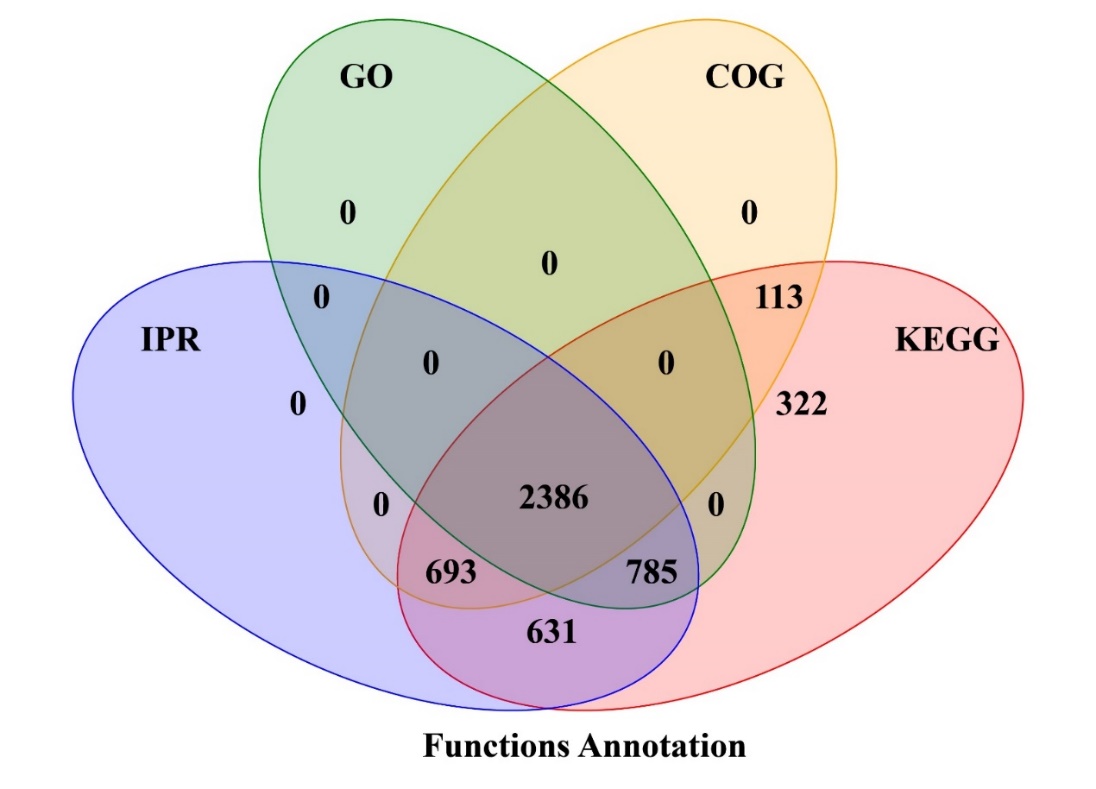


**Figure S4 Venn diagram of the number of overlapping proteins annotated in the IPR, GO, GOG, KEGG database for six citrus vacuolar proteomes.**

A total of 4930 proteins were annotated, which are listed in Table S1.


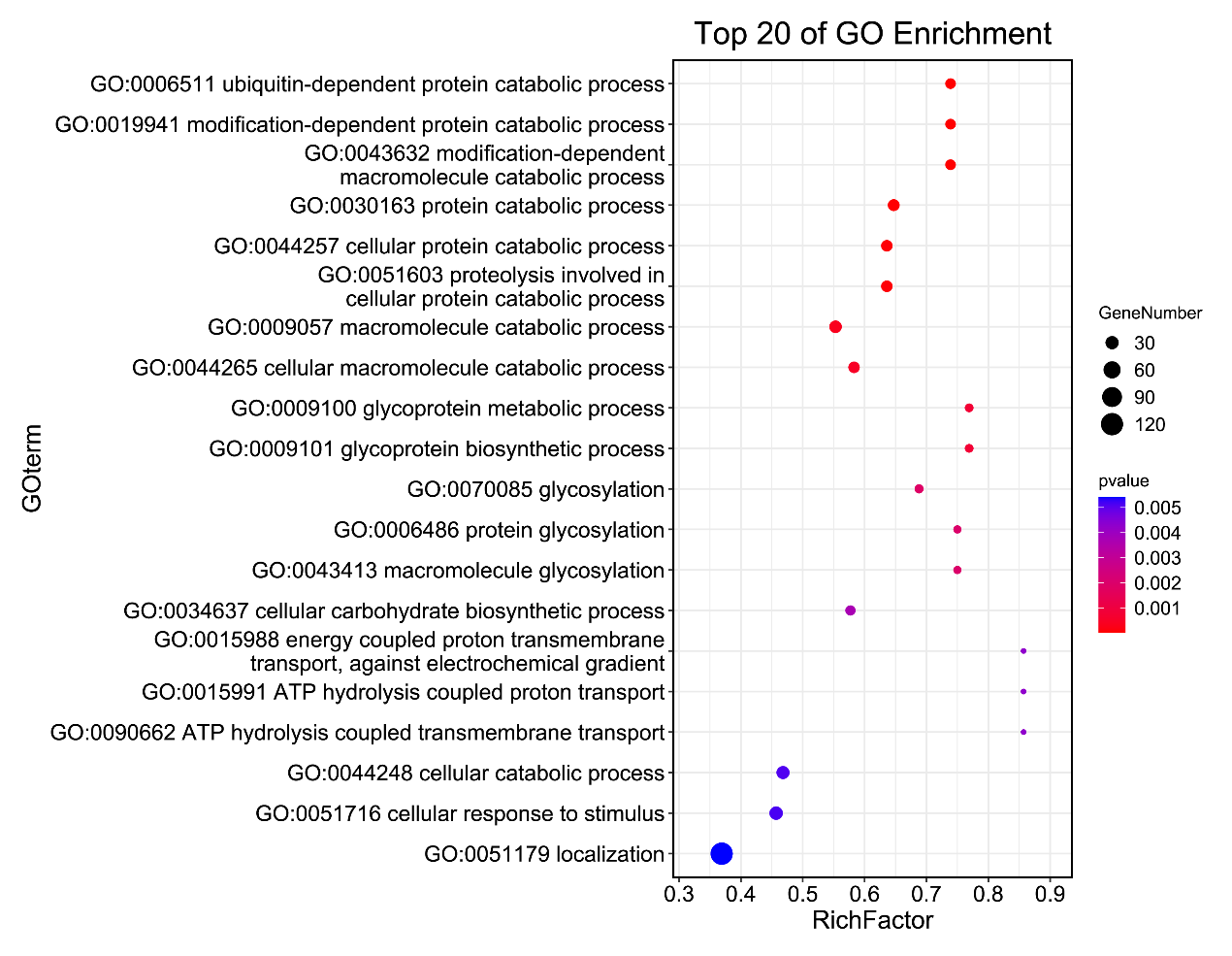


**Figure S5 GO functional enrichment of 1443 proteins shared by six citrus vacuolar samples.**


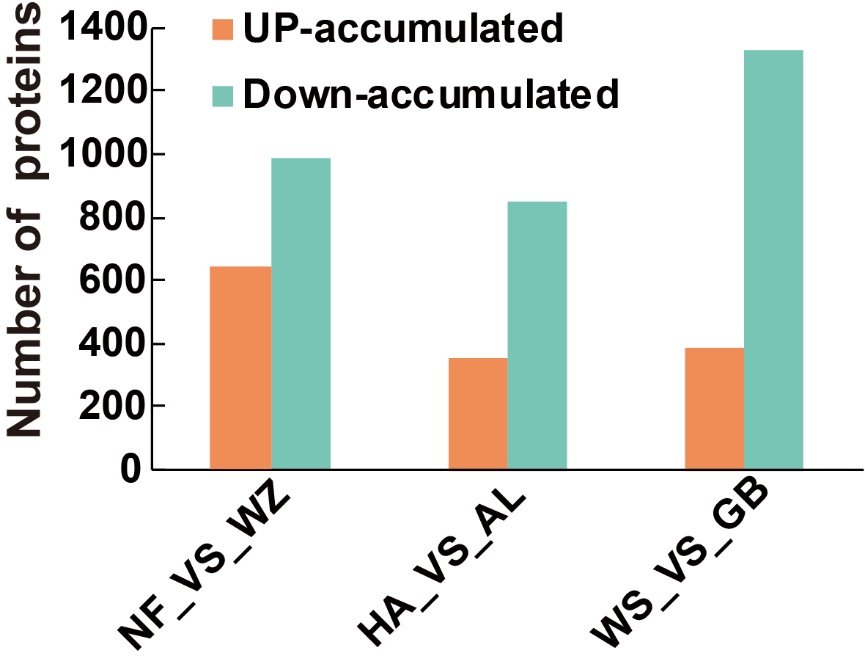


**Figure S6 Number of differentially expressed proteins (DAPs) between three citrus groups.**

NF vs WZ; HA vs AL; WS vs GB

**
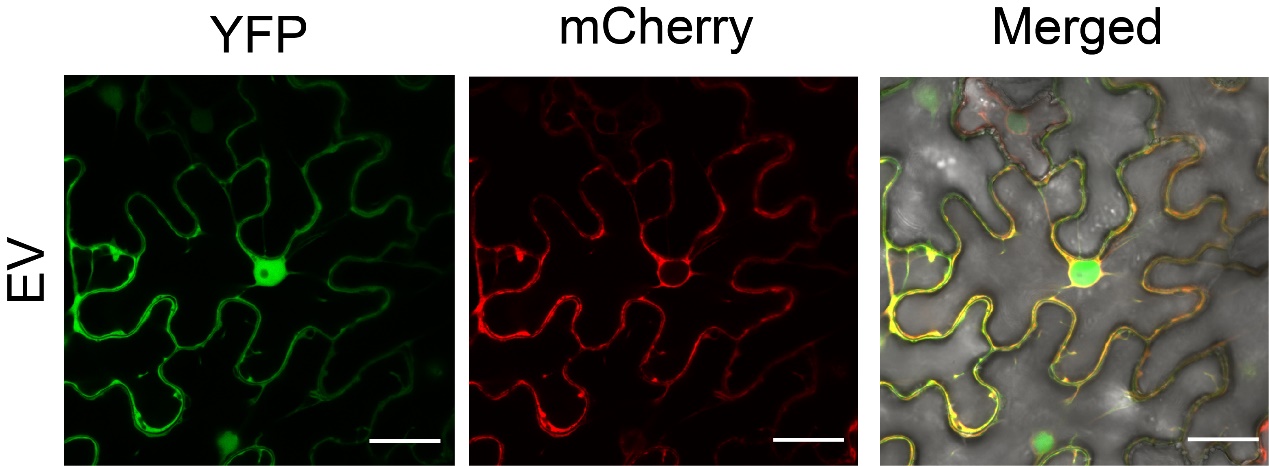
**

**Figure S7 Co-localization of empty vector and tonoplast mCherry-marker in tobacco epidermal cells.**

Scale bars = 25 μm.
